# The Effects of Hydrophobicity and Textural Properties on Hexamethyldisiloxane Adsorption in Reduced Graphene Oxide Aerogels

**DOI:** 10.3390/molecules26041130

**Published:** 2021-02-20

**Authors:** Xifeng Hou, Yanhui Zheng, Xiaolong Ma, Yuheng Liu, Zichuan Ma

**Affiliations:** 1 Hebei Key Laboratory of Inorganic Nano-Materilas, College of Chemistry and Material Science, Hebei Normal University, Shijiazhuang 050024, Hebei, China; xifenghoucc@163.com (X.H.); zhengyh0308@163.com (Y.Z.); 2 Shijiazhuang Vocational College of Finance & Economics, Shijiazhuang 050061, Hebei, China; 3 School of Environmental Science and Engineering, Hebei University of Science and Technology, Shijiazhuang 050018, Hebei, China; maxiaolong2410@163.com; 4 College of Pharmaceutical Sciences, Hebei Medical University, Shijiazhuang 050017, Hebei, China; liu2795478@163.com

**Keywords:** reduced graphene oxide aerogel, hexamethyldisiloxane, adsorption, hydrophobicity, siloxane

## Abstract

To expand the applications of graphene-based materials to biogas purification, a series of reduced graphene oxide aerogels (rGOAs) were prepared from industrial grade graphene oxide using a simple hydrothermal method. The influences of the hydrothermal preparation temperature on the textural properties, hydrophobicity and physisorption behavior of the rGOAs were investigated using a range of physical and spectroscopic techniques. The results showed that the rGOAs had a macro-porous three-dimensional network structure. Raising the hydrothermal treatment temperature reduced the number of oxygen-containing groups, whereas the specific surface area (*S*_BET_), micropore volume (*V*_micro_) and water contact angle values of the rGOAs all increased. The dynamic adsorption properties of the rGOAs towards hexamethyldisiloxane (L2) increased with increasing hydrothermal treatment temperature and the breakthrough adsorption capacity showed a significant linear association with *S*_BET_, *V*_micro_ and contact angle. There was a significant negative association between the breakthrough time and inlet concentration of L2, and the relationship could be reliably predicted with a simple empirical formula. L2 adsorption also increased with decreasing bed temperature. Saturated rGOAs were readily regenerated by a brief heat-treatment at 100 °C. This study has demonstrated the potential of novel rGOA for applications using adsorbents to remove siloxanes from biogas.

## 1. Introduction

Biogas is an alternative energy source produced by the anaerobic digestion of organic material. It can be produced from raw materials such as agricultural, food and municipal waste products, and sewage sludge [1,2]. Due to its relatively high methane content and calorific value, biogas has been widely investigated as a renewable energy source for heating and power generation [3,4]. However, siloxanes, present at low concentrations in biogas, can compromise the operation of biogas-to-energy facilities, presenting technical challenges for its widespread use [5]. During combustion, siloxanes such as hexamethyldisiloxane (L2) and octamethylcyclotetrasiloxane (D4) can form white deposits (SiO_2_) on critical components, decreasing their performance and increasing the costs of maintenance and operation [6,7]. Consequently, amounts of siloxanes in biogas must be reduced below threshold levels (e.g., 10 mg siloxane/m^3^ of methane) prior to use in energy applications [8,9,10].

Materials currently used to reduce siloxanes in biogas include high efficiency adsorbents such as activated carbon, silica and molecular sieves [5,11,12,13]. While these adsorbents are relatively low cost, simple to use [14,15,16] and high in adsorption capacity, their adsorption cycle performances are limited, restricting their practical applications. Previously, we developed a modified silica gel siloxane adsorbent with good recyclability and found that its adsorption performance was affected by its textural properties, such as the specific surface area (*S*_BET_), total pore volume (*V*_tot_), micropore volume (*V*_micro_) and contact angle [16,17,18]. Reduced graphene oxide aerogels (rGOAs) have good textural properties and strong hydrophobicity [19,20,21,22,23,24,25,26], although their application to biogas siloxane removal has not been reported. In addition, rGOAs were prepared using commercially available industrial grade graphene oxide (IGGO) without any reducing agents. Compared with traditional preparation methods [25], this strategy is simple, low cost, flexible, versatile, readily scalable and insensitive to environmental conditions.

Hence, the aims of this study were to develop a novel siloxane adsorbent based on readily obtained rGOAs for the purification of biogas, and determine the key factors affecting its performance. An understanding of these factors will assist the future design of rGOAs for gas phase dynamic adsorption applications.

## 2. Results and Discussion

### 2.1. Effects of Hydrothermal Temperature on Texture Properties and Hydrophobicity

**Figure 1:**
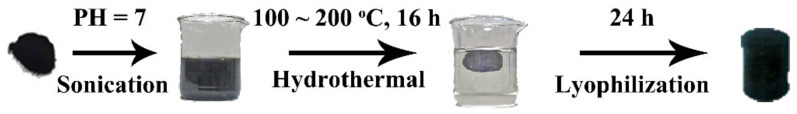
Illustration of the preparation process of the reduced graphene oxide aerogel (rGOA).

**Figure 2:**
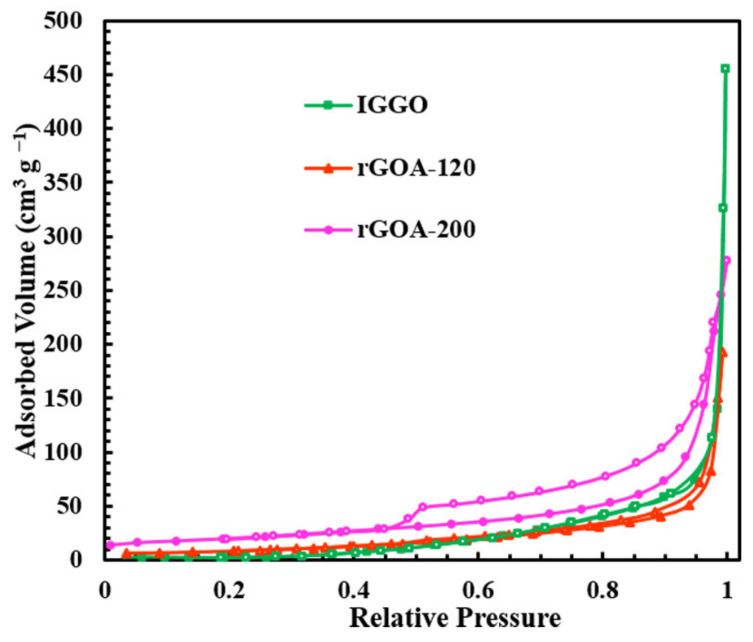
N_2_ adsorption–desorption isotherms.

The hydrothermal preparation process is illustrated in Figure 1 for rGOA-200. The procedure involved three main steps: (1) sonication to obtain a 10 mg·L^−1^ IGGO precursor dispersion (pH = 7.0); (2) partial hydrothermal reduction of oxygen-containing functional groups distributed on the IGGO surface and promotion of rGO sheet self-assembly into the rGO hydrogel; (3) lyophilization to minimize the capillary force and achieve the rGOA three-dimensional (3D) network structure [27,28,29]. The hydrothermal synthesis temperature was believed to be a significant factor affecting the textural properties and hydrophobicity of rGOA materials [27,30,31,32]. Figure 2 shows the N_2_ adsorption–desorption isotherms of IGGO and two representative rGOA composites (i.e., rGOA-120 and rGOA-200). The calculated textural parameters from the N_2_ physisorption measurements of these materials are listed in Table 1. The shapes of the gas adsorption isotherms for rGOAs were consistent with IUPAC type V curves with a type H3 hysteresis loop [8,33]. The shapes of N_2_ adsorption-desorption isotherms were slightly increased at low relative pressures (*P*/*P*_0_ < 0.4) and sharply increased at high relative pressure (0.93 < *P*/*P*_0_ < 1.0), indicating the coexistence of slotted mesopores and macropores [16,34]. Compared with rGOAs, IGGO had a significantly smaller N_2_ adsorption volume and exhibited a type III isotherm, reflecting its relatively non-porous/macroporous structure [35].

Table 1 shows that D_aver_ was reduced while *S*_BET_ and *V*_micro_ both increased with increasing hydrothermal temperature. This monotonic behavior was not observed for *V*_tot_, which reached a maximum value of 0.45 cm^3^·g^−1^ for rGOA-140, indicating that the 3D macroscopic assemblies of rGOA had a mesoporous/macroporous texture [16,22].

**Figure 3:**
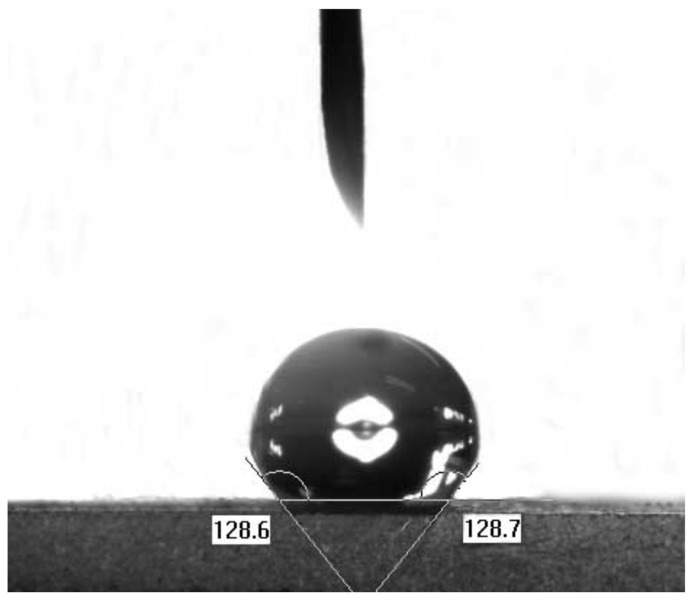
Image showing a water droplet on the surface of an rGOA-200 film.

Figure 3 shows the contact angle analysis diagram for rGOA-200 demonstrating a hydrophobic surface [36,37]. The contact angle, and hence the hydrophobicity, increased with increasing hydrothermal temperature for all rGOAs (see Table 1).

### 2.2. XRD, SEM, TEM, Raman, Elemental and FTIR Analysis of rGOAs and IGGO

**Figure 4:**
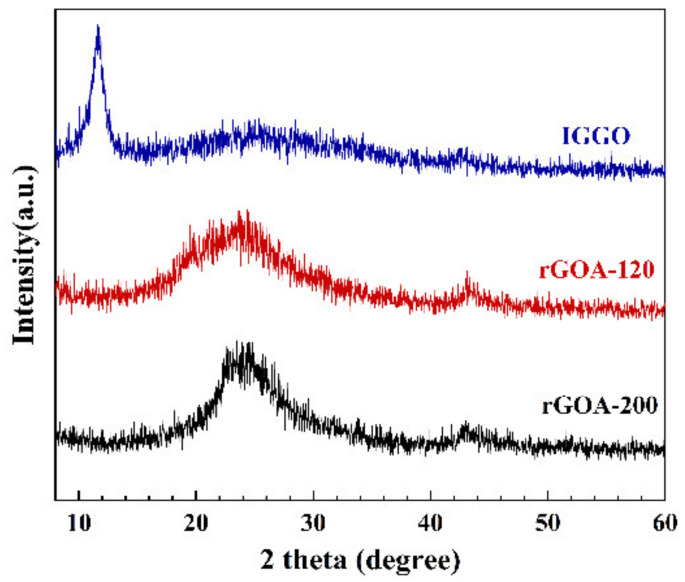
XRD patterns of the materials.

Figure 4 shows the XRD patterns obtained from IGGO, rGOA-120 and rGOA-200. The sharp peak at 2θ = 11.6° (IGGO), corresponding to the (001) plane with an interlayer spacing (d-spacing) of 0.76 nm, was typical of the separation between the layered IGGO sheets [38]. The absence of this peak for both rGOA-120 and rGOA-200, and the appearance of a new peak at ≈24° (d_002_ of ca. 0.37 nm), are both consistent with the combining of graphene sheet structures during a hydrothermal reduction [30,39].

**Figure 5:**
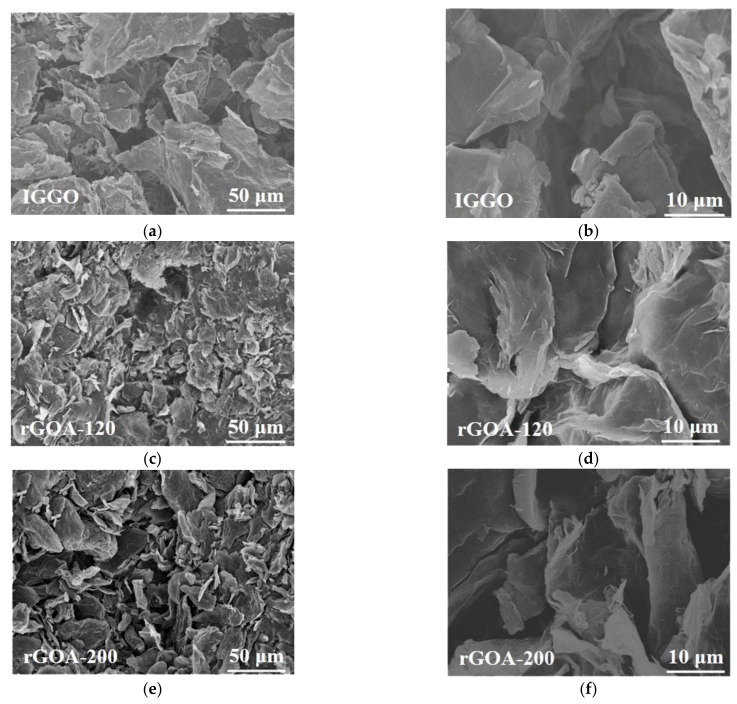
SEM images of IGGO (**a**,**b**), rGOA-120(**c**,**d**) and rGOA-200 (**e**,**f**).

**Figure 6:**
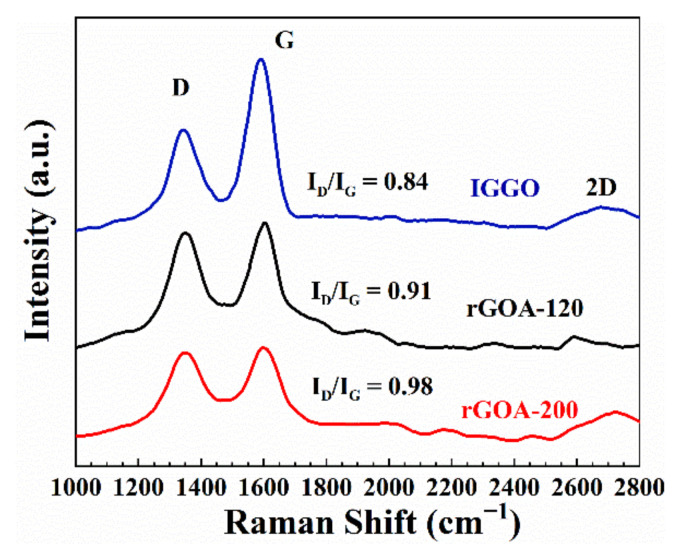
Raman spectra of IGGO, rGOA-120 and rGOA-200.

**Figure 7:**
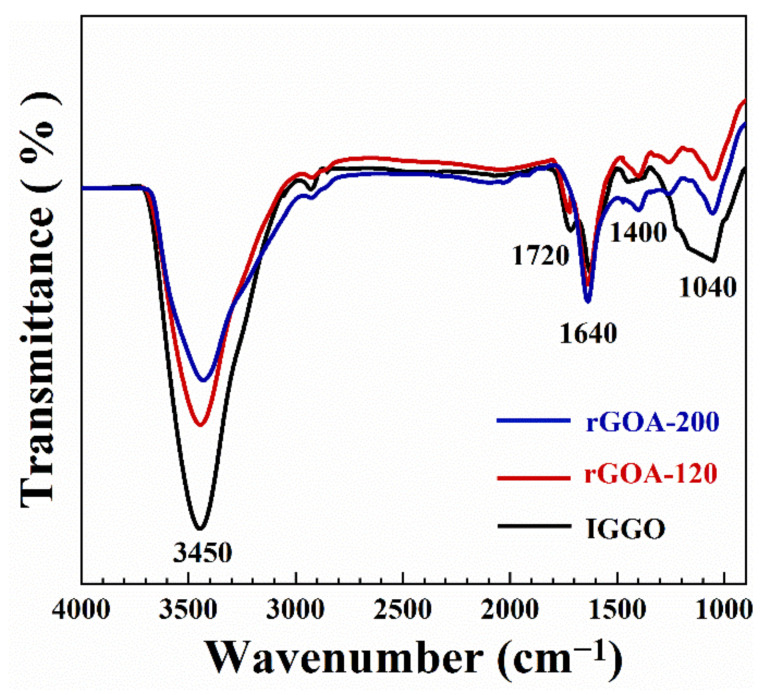
FTIR spectra of IGGO, rGOA-120 and rGOA-200.

The SEM images of IGGO and the two typical rGOA samples are given in Figure 5a–f. Compared with IGGO, the rGOAs exhibited a rich macroporous 3D network structure and more slit-like pores. Furthermore, TEM images of IGGO and rGOA-200 (Figure S1, Supplementary Materials) illustrate the overlapping of transparent graphene nanosheets with many wrinkles. IGGO had overlaps of multiple layers, whereas rGOA-200 exhibited only a few layers after hydrothermal reduction. This morphology is also consistent with the N_2_ adsorption-desorption isotherm results. The Raman spectra of IGGO and the two typical rGOA samples are given in Figure 6. The peaks located in the range of 2600–2800 cm^−1^ are the 2D bands, which are another characteristic peak of graphene [40]. The rGOA showed a fairly broad and up-shift 2D peak in the Raman spectrum, indicating its few-layer structure, which was consistent with the TEM results. The increased ratio of the I_D_/I_G_ bands for rGOA-120 and rGOA-200 indicated that these structures were more disordered compared to IGGO [37,40]. In general, the characteristic D (1350 cm^−1^) and G (1590 cm^−1^) bands in the Raman spectra of graphite-based materials can be attributed to the lattice defects and the in-plane stretching vibrations of sp^2^ hybridized atoms [41]. The increase in I_D_/I_G_ from IGGO → rGOA was also consistent with removal of the oxygen-containing moieties present in IGGO, and their removal was temperature dependent. These observations can be further confirmed by the element analysis results (Table S1, Supplementary Materials). The oxygen content in both rGOA-120 and rGOA-200 materials was significantly diminished in comparison with IGGO, and rGOA-200 had less oxygen content than rGOA-120. The changes to oxygen-containing groups in IGGO with hydrothermal temperature treatment could be observed in the normalized FTIR spectra of IGGO, rGOA-120 and rGOA-200 shown in Figure 7. The intensities of the stretching vibration peaks at 1720 cm^−1^ (carbonyl and carboxyl groups) and peaks at 3450 cm^−1^ (-OH stretching vibration) and 1400 cm^−1^ (-OH bending vibration) all decreased in the order IGGO > rGOA-120 > rGOA-200 [19]. Similarly, the characteristic band at 1040 cm^−1^ (C–O bending vibration) weakened in the order IGGO > rGOA-120 > rGOA-200, indicating partial cleavage of C–O bonds during the hydrothermal reduction. In addition, the intensities of the characteristic stretching vibrations at 1640 cm^−1^ (C=C group) for both rGOA samples were significantly increased [42], which is indicative of the restoration of the π-conjugation network of graphene following a hydrothermal reduction [43].

### 2.3. Comparison of the Dynamic Adsorption Performances of Different rGOAs

L2 was chosen as a representative siloxane impurity in biogas to assess the adsorption and regeneration performances of the adsorbents.

**Figure 8:**
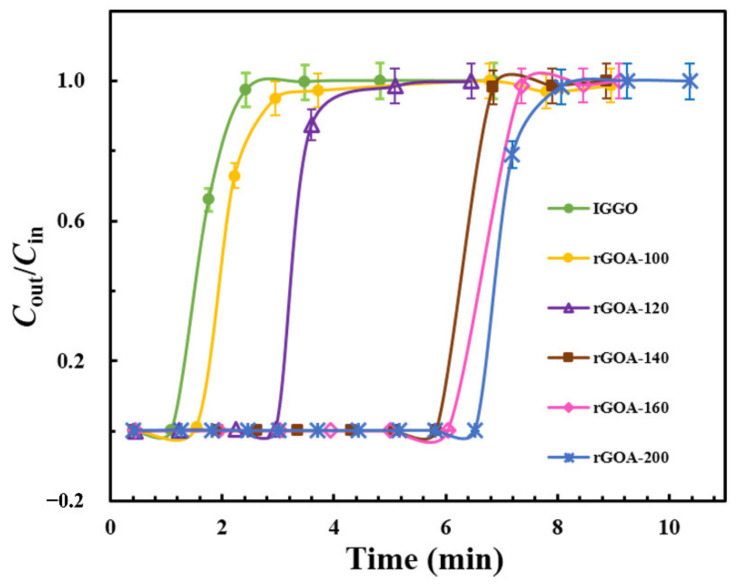
Breakthrough curves of the adsorbents for L2.

**Figure 9:**
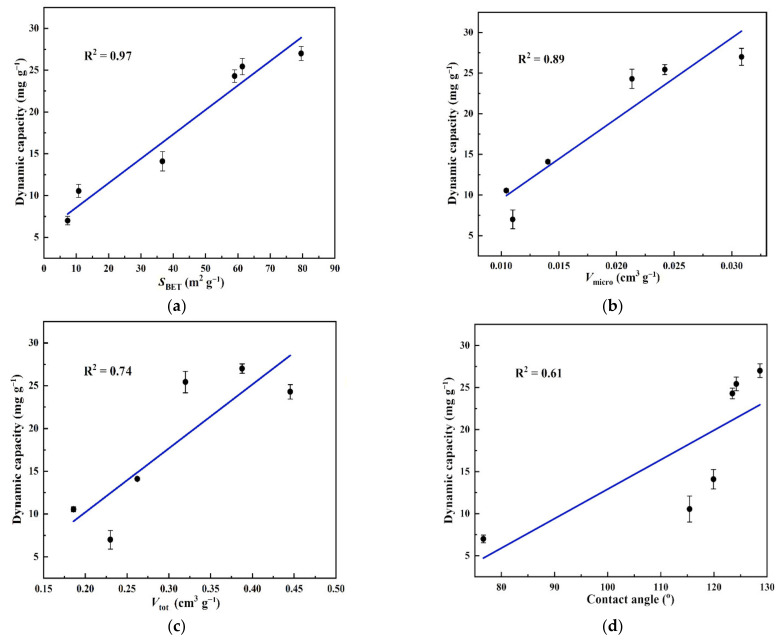
Relationships between *Q*_B_ and *S*_BET_ (**a**), *Q*_B_ and *V*_tot_ (**b**), *Q*_B_ and *V*_micro_ (**c**) and *Q*_B_ and contact angle (**d**) for the six adsorbents.

Figure 8 shows the adsorption breakthrough curves obtained at 20 °C for L2 with IGGO and the five rGOA materials (the inlet concentration: *C*_in_ = 38.3 mg·L^−1^; the gas flow rate: *V*_q_ = 0.01 L·min^−1^). The corresponding dynamic adsorption parameters (*t*_B_, *Q*_B_ and *Q*_m_) calculated from these isotherm curves are given in Table 2. Compared with IGGO, which had a very low removal efficiency for L2, the breakthrough curves obtained with each rGOA were progressively shifted towards increased time with increasing hydrothermal temperature. Consequently, the parameters *t*_B_, *Q*_B_ and *Q*_m_ also increased with increasing temperature, with rGOA-200 demonstrating the highest adsorption capacity (*t*_B_ = 6.5 min; *Q*_B_ = 24.7 mg·g^−1^; *Q*_m_ = 27.4 mg·g^−1^). These trends were consistent with those obtained from the N_2_ physisorption measurements (*S*_BET_, *V*_micro_) and contact angle analysis of the adsorbents (see Table 1). Figure 9a–d show the relationships between *Q*_B_ and the surface texture/hydrophobicity parameters *S*_BET_, *V*_micro_, *V*_tot_ and contact angle for the six adsorbents, respectively. Inspection of the linear correlation coefficients (R^2^) for each fitted line showed that *S*_BET_ (R^2^ = 0.97; *p* < 0.05) and *V*_micro_ (R^2^ = 0.89; *p* < 0.05) were significantly associated with *Q*_B_, indicating that these were important parameters for the adsorption of L2. Since *S*_BET_ is theoretically dependent on the abundance of micropores/mesopores in the structure, it may be inferred that the mechanism of adsorption of L2 on rGOAs may be dependent on capillary condensation and hydrophobic effects [16,17,18].

### 2.4. Influences of Process Conditions on the Adsorption Performance of rGOA-200

**Figure 10:**
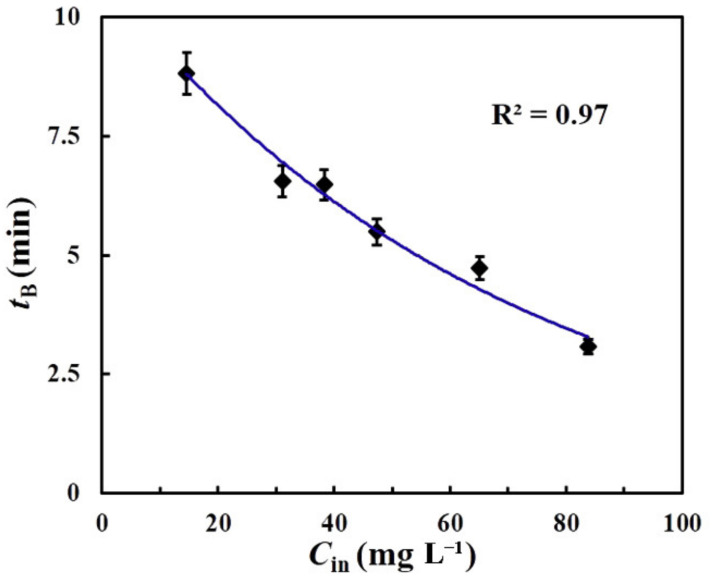
The effect of inlet concentration on breakthrough time.

An understanding of the correlation between the breakthrough time and inlet concentration is important for the industrial application of adsorbents [12,14,18,44]. Figure 10 shows the experimental relationship between *t*_B_ and *C*_in_ from the adsorption of L2 by rGOA-200 and the simulated curve fitting the experimental data. The results showed that there was a significant negative association between *t*_B_ and *C*_in_ (R^2^ = 0.97, *p* < 0.05), and the relationship could be reliably predicted by Equation (1). This provided a theoretical basis for potential industrial applications involving siloxane purification.

(1) $$t_{B}\;=\;10.836{\;\times \;e}^{-\;\frac{C_{\mathrm{in}}}{71}}$$

The influence of bed temperature on L2 adsorption by rGOA-200 is given in Table 3. As the temperature decreased, *t*_B_, *Q*_B_ and *Q*_m_ increased, demonstrating that siloxane adsorption performance by rGOA-200 could be attained at low temperatures. This observation could be explained by the exothermic micropore filling and hydrophobic effects of the rGOA materials [22,28,45]. Hence, lowering the temperature would promote both mechanisms.

### 2.5. Recycling Performance of rGOA-200

**Figure 11:**
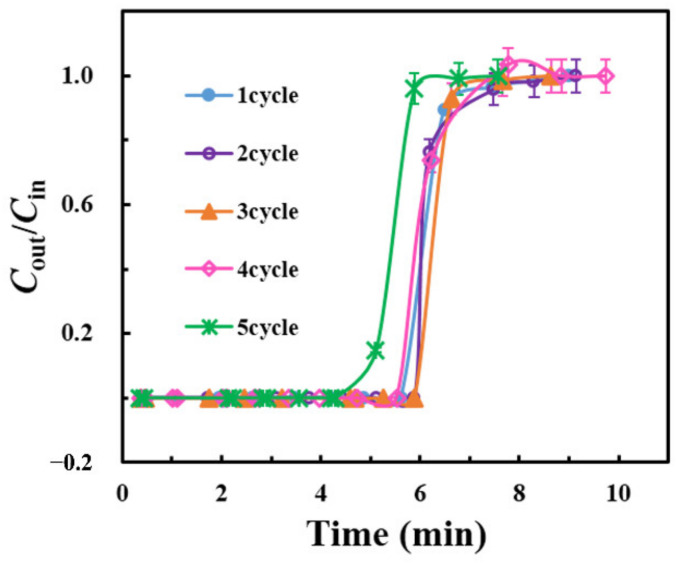
Adsorption breakthrough curves for L2 of rGOA-200 in the repeated cycles.

L2 saturated rGOA-200 adsorbent could be regenerated by a simple heat-treatment at 100 °C for 30 min. Figure 11 shows the results obtained from five L2 adsorption-desorption cycles, and Figure S2 shows that there was no residual L2 on the rGOA-200 after continuous cycles. The performance of rGOA-200 was almost consistent after each cycle, indicating that it had good reusability, mainly due to the physical adsorption mechanism. As can be seen from Table 4, although the adsorption capacity of rGOA remained lower than those of several carbon-based materials, its regeneration performance was the highest, with a more than 99% regeneration efficiency.

## 3. Experimental Section

### 3.1. Materials

All reagents were purchased from commercial suppliers and used without further purification. IGGO was obtained from Hengqiu Graphene Technology Co., Ltd. (Suzhou, China). Hydrochloric acid (36 wt%) was from Beijing Chemical Reagent Company (Beijing, China). Tert-butanol (99.7 wt%) was from Yongda Chemical Co., Ltd. (Shandong, China). L2 (99 wt%) was from Aladdin (Shanghai, China).

### 3.2. Preparation of rGOA Adsorbents

IGGO powder (0.6 g) was dispersed in hydrochloric acid (60 mL of 0.1 mol·L^−1^) for 1 h, washed thoroughly (deionized water) and reconstituted in deionized water to obtain a 10 mg·mL^−1^ dispersion. The IGGO dispersion was then sonicated for 1 h prior to sealing aliquots (60 mL) into polytetrafluoroethylene lined containers (80 mL) and subjecting them to hydrothermal treatments at 80, 100, 120, 140, 160 and 200 °C for 16 h in a thermostatic oven [36]. IGGO dispersions formed hydrogels after hydrothermal treatments of >80 °C. To protect the pore structure of the hydrogel, the material was soaked in *tert*-butanol for 6 h and pre-frozen prior to lyophilization (< 20 Pa, 24 h) [49,50,51]. The resultant adsorbents were labelled rGOA-x, where x represented the hydrothermal temperature.

### 3.3. Characterization of IGGO and rGOA-x

Surface morphology analysis of the adsorbents was performed using a S-4800 scanning electron microscope (SEM; Hitachi, Tokyo, Japan) and a transmission electron microscope (TEM, Hitachi, Tokyo, Japan) operated at 120 kV. The N_2_ adsorption-desorption isotherms were obtained from out-gassed samples (95 °C for 12 h) at −196 °C using a Kubo ×1000 high-performance micropore analyzer (Beijing Builder Co., Ltd., Beijing, China) with the precision of 0.001 m^2^·g^−1^. *S*_BET_, *V*_tot_ and *V*_micro_ were calculated according to the Brunauer-Emmett-Teller (BET) and Dubinin-Radushkevich equations. The D_aver_ was obtained as 4*V*_tot_/*S*_BET_, based on the BET method at a relative pressure (*P*/*P*_0_) range of 10^−5^ < *P*/*P*_0_ < 0.98 [52]. The X-ray diffraction (XRD) spectra were obtained using a D8 Advance X-ray diffractometer (Brucker AXS, Karlsruhe, Germany). Raman spectra were obtained with a 514 nm excitation wavelength on an inVia™ Raman spectrometer (Renishaw, New Mills, UK). Elemental analyses of C, H, and N was performed on a Vario EL III elemental analyzer (Elementar, Langenselbold, Germany). The Fourier transform infrared (FTIR) spectra (KBr pellet method) were acquired on a Nicolet 6700 spectrometer (Theromo Fisher, Waltham, MA, USA). The contact angle was obtained from a water drop (3 μL) at room temperature using a JY-PHb contact angle/interface system (Jinhe Instrumentation, Nanjing, Jiangsu, China) and the sample was uniformly placed in the edge of the glass substrate with the thickness < 0.1 mm. L2 concentration in the gas stream was measured using a 9790 gas chromatography system equipped with a flame ionization detector (GC-FID; Zhejiang Fuli Analytical Instrument Co., Ltd., Wenling, Zhejiang, China). Separation was carried out isothermally (200 °C) on a 2.0 m × 2.0 mm I.D. column packed with poly divinylbenzene porous beads (GDX-102, Aladdin, Shanghai, China) using N_2_ carrier gas (30 mL·min^−1^).

### 3.4. L2 Adsorption-Desorption Behavior

Dynamic gas adsorption tests were carried out on 0.1 g of adsorbent with 0.2 g of pine chips mixed in evenly at 20 °C; the inlet concentration of L2 was 38.28 mg·L^−1^ at a total gas flow rate of 0.01 L·min^−1^. The system was operated until the outlet concentration (*C*_out,*t*_) was equal to the inlet concentration (*C*_in_), i.e., when the system had attained adsorption saturation, and the corresponding breakthrough curves were obtained by plotting *C*_out,*t*_/*C*_in_ versus *t*. Adsorbent performance was evaluated by the following indicators: (i) breakthrough time (*t*_B_, min), defined by *C*_out,*t*_/*C*_in_ = 0.05; (ii) the L2 breakthrough adsorption capacity at *t*_B_ (*Q*_B_, mg·g^−1^); and (iii) the L2 adsorption capacity at saturation (*Q*_m_, mg·g^−1^). *Q*_B_ and *Q*_m_ were calculated from Equation (2) [16]:

(2) $$Q_{t}=\frac{V_{q}C_{\mathrm{in}}}{m}\int_{0}^{t}\left(1-\frac{C_{\mathrm{out},t}}{C_{\mathrm{in}}}\right)dt,$$

where *V*_q_ was the gas flow rate (L·min^−1^), *m* was the adsorbent weight (g), *C*_in_ was the inlet concentration (mg·L^−1^) and *C*_out,*t*_ was the outlet concentration (mg·L^−1^) at an adsorption time *t* (min). According to this equation, a fixed time point *t* corresponded to a unique *Q_t_*. When *t* was equal to *t*_B_, *Q*_B_ could be obtained. Similarly, when *t* corresponded to the saturation point, i.e., where *C*_out,*t*_/*C*_in_ ≈ 1, *Q*_m_ could be obtained.

Desorption characteristics were determined by heating the saturated sample in situ at 100 °C for 30 min under continuous N_2_ purge and monitoring the residual concentration of L2 by GC-FID. A total of five adsorption-desorption cycles were carried out to determine the recycling performance of the adsorbent.

## 4. Conclusions

A series of hydrophobic and porous rGOA materials were successfully prepared from IGGO using a simple hydrothermal method. Surface morphology analysis showed that the rGOAs had a macroporus 3D network structure. Spectroscopic analysis (XRD, FTIR, Raman) confirmed a non-crystalline nature and attenuation of oxygen-containing groups following the reduction of IGGO to rGOAs. The *S*_BET_, *V*_micro_ and water contact angle of each rGOA increased with increasing hydrothermal temperature treatment of IGGO. Adsorption breakthrough experiments revealed that the adsorptive performance of the rGOAs for L2 increased with increasing hydrothermal treatment temperature. *Q*_B_ showed a significant linear association with *S*_BET_ (R^2^ = 0.97, *p* < 0.05) and *V*_micro_ (R^2^ = 0.89, *p* < 0.05), indicating that they were important parameters for the adsorption of L2. rGOA-200 exhibited the highest adsorption of all rGOAs towards L2 at 20 °C (*t*_B_ = 6.5 min, *Q*_B_ = 24.7 mg·g^−1^, *Q*_m_ = 27.4 mg·g^−1^). Values of *t*_B_, *Q*_B_ and *Q*_m_ all increased with decreasing bed temperature. Saturated rGOA-200 was readily regenerated by a brief heat-treatment at 100 °C.

## Figures and Tables

**Table 1:** Textural properties and contact angles of the materials.

Adsorbent	*S*_BET_, m^2^·g^−1^	*V*_micro_, cm^3^·g^−1^	*V*_tot_, cm^3^·g^−1^	D_aver_, nm	Contact Angle, °
IGGO	7.4	0.011	0.23	124.3	76.6 ± 0.4
rGOA-100	10.7	0.010	0.19	71.0	118.1 ± 0.2
rGOA-120	36.7	0.014	0.26	28.3	119.9 ± 0.3
rGOA-140	59.0	0.021	0.45	30.5	123.5 ± 0.3
rGOA-160	61.4	0.024	0.32	20.9	124.2 ± 0.3
rGOA-200	79.6	0.031	0.39	19.6	128.7 ± 0.6

**Table 2:** Adsorption parameters of the adsorbents for L2.

Adsorbent	*t*_B_^1^, min	*Q*_B_^2^, mg·g^−1^	*Q*_m_^3^, mg·g^−1^
IGGO	1.1	4.4	7.1
rGOA-100	1.8	7.2	10.5
rGOA-120	3.0	12.3	14.1
rGOA-140	5.8	22.5	24.3
rGOA-160	6.0	23.1	25.4
rGOA-200	6.5	24.7	27.4

^1^ Breakthrough time (*t*_B_, min). ^2^ The L2 breakthrough adsorption capacity at *t*_B_ (*Q*_B_, mg·g^−1^). ^3^ The L2 adsorption capacity at saturation (*Q*_m_, mg·g^−1^).

**Table 3:** Adsorption properties of rGOA-200 at different temperatures.

Entry	Temp., °C	*t*_B_, min	*Q*_B_, mg·g^−1^	*Q*_m_, mg·g^−1^
1	0	8.0	30.1	31.8
2	10	6.5	24.6	26.0
3	20	5.6	21.3	22.9
4	30	4.9	18.6	20.0
5	40	4.9	18.4	19.0
6	50	4.5	17.3	18.8

**Table 4:** Adsorption and regeneration capacities of different carbon materials for siloxanes.

Adsorbent	*Q*_m_, mg·g^−1^	RE ^a^, %	Regeneration Method	Reference
Activated carbons	10–100	70–80	Four-step heating treatment at 80–160 °C	[46]
Activated carbons	60–878	50	Heating at 100–200 °C	[47]
Activated carbons	526	40–92	Advanced oxidation processes	[48]
rGOA-200	32	>99	Heating at 100 °C	This work

^a^ Regeneration efficiency after 1 cycle (RE).

## Data Availability

Data is contained within the article or supplementary materials. The data presented in this study are available in Supplementary Materials.

## Supplementary Materials

The following are available online, Figure S1: TEM images of rGOA-200 and IGGO, Figure S2: FTIR spectra of samples processed by different treatments, Table S1: Elemental contents of IGGO, rGOA-120, rGOA-200.

## Sample Availability

Samples of the compounds IGGO and rGOAs are available from the authors.
