# Supplementary material for: The Effects of Hydrophobicity and Textural Properties on Hexamethyldisiloxane Adsorption in Reduced Graphene Oxide Aerogels
Source: Molecules. 2021 Feb 20;26(4):1130. doi: 10.3390/molecules26041130 (PMC7924388; doi:10.3390/molecules26041130)
Supplement: Supplementary file 1 [file molecules-26-01130-s001.pdf]

molecules

MDPI

Supplementary Material

Effect of Hydrophobicity and Textural Properties on Hexamethyldisiloxane Adsorption in Reduced Graphene Oxide Aerogels

Xifeng Hou [1], Yanhui Zheng[1,2], Xiaolong Ma [3], Yuheng Liu [4], and Zichuan Ma [1,*]

1 Hebei Key Laboratory of Inorganic Nano-materilas, College of Chemistry and Material Science, Hebei Normal University, Shijiazhuang 050024, Hebei, PR China; xifenghoucc@163.com (X. H.); zhengyh0308@163.com (Y. Z.); mazc@hebtu.edu.cn (Z. M.);
2 Shijiazhuang Vocational College of Finance & Economics, Shijiazhuang 050061, Hebei, PR China; zhengyh0308@163.com (Y. Z.);
3 School of Environmental Science and Engineering, Hebei University of Science and Technology, Shijiazhuang 050018, Hebei, PR China; maxiaolong2410@163.com (X. M.);
4 College of Pharmaceutical Sciences, Hebei Medical University, Shijiazhuang 050017, Hebei, PR China; liu2795478@163.com (Y. L.);
* Correspondence: mazc@hebtu.edu.cn; Tel.: +86 311 80787400

Contents

Table S1. Elemental contents of IGGO, rGOA-120, rGOA-200..1
Figure S1. TEM images of rGOA-200 and IGGO..2
Figure S2. FTIR spectra of samples processed by different treatments...2

Table S1. Elemental contents of IGGO, rGOA-120, rGOA-200.

Samples		IGGO	rGOA-120	rGOA-200
Content, wt.%	C	49.98	79.63	81.49
	H	2.37	1.12	1.03
	O [a]	47.65	19.23	17.47
	N	-	0.02	0.01

[a] Obtained by subtracting the content of the other three elements by 100%.
- Not detected.

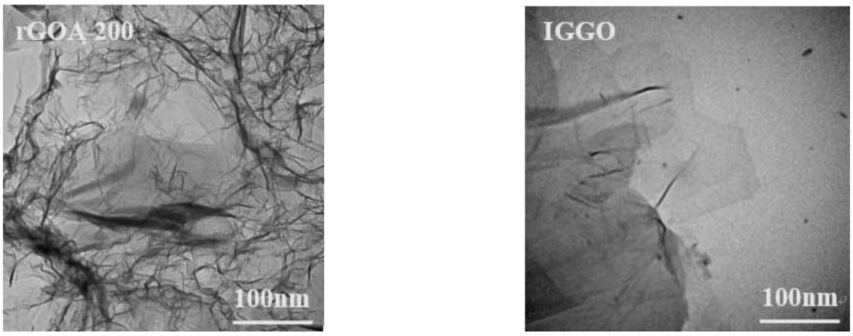

(a) (b)

Figure S1. TEM images of rGOA-200 and IGGO.

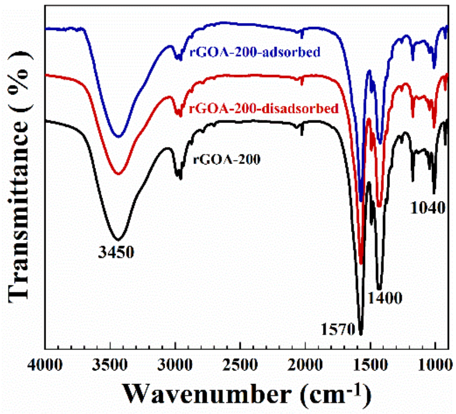

Figure S2. FTIR spectra of samples processed by different treatments.
